# Extra-Intestinal Effects of *C.* *difficile* Toxin A and B: An In Vivo Study Using the Zebrafish Embryo Model

**DOI:** 10.3390/cells9122575

**Published:** 2020-12-01

**Authors:** Federica Tonon, Stefano Di Bella, Gabriele Grassi, Roberto Luzzati, Paolo Ascenzi, Alessandra di Masi, Cristina Zennaro

**Affiliations:** 1 Department of Medical, Surgery and Health Sciences, University of Trieste, 34149 Trieste, Italy; stefano932@gmail.com (S.D.B.); roberto.luzzati@asugi.sanita.fvg.it (R.L.); cristina.zennaro@asugi.sanita.fvg.it (C.Z.); 2 Department of Life Sciences, University of Trieste, 34128 Trieste, Italy; ggrassi@units.it; 3 Department of Sciences, Roma Tre University, 00154 Rome, Italy; ascenzi@uniroma3.it (P.A.); alessandra.dimasi@uniroma3.it (A.d.M.)

**Keywords:** zebrafish, toxins, *C.difficile* infection

## Abstract

*C.difficile* infection (CDI) is not a merely “gut-confined” disease as toxemia could drive the development of CDI-related extra-intestinal effects. These effects could explain the high CDI-associated mortality, not just justified by diarrhea and dehydration. Here, the extra-intestinal effects of toxin A (TcdA) and B (TcdB) produced by *C. difficile* have been studied in vivo using the zebrafish embryo model. Noteworthy, protective properties of human serum albumin (HSA) towards toxins-induced extra-intestinal effects were also addressed. Zebrafish embryos were treated with TcdA, TcdB and/or HSA at 24 h post-fertilization. Embryos were analyzed for 48 h after treatment to check vital signs and morphological changes. Markers related to cardio-vascular damage and inflammation were evaluated by Real-Time quantitative PCR and/or western blotting. Both toxins induced cardiovascular damage in zebrafish embryos by different mechanisms: (i) direct toxicity (i.e., pericardial edema, cardiac chambers enlargement, endothelial alteration); (ii) increased hormonal production and release (i.e., atrial natriuretic peptide (ANP) and brain natriuretic peptide (BNP)), (iii) alteration of the vascular system through the increase of the vascular endothelial growth factor (VEGF-A) levels, as well as of its receptors, (iv) pro-inflammatory response through high cytokines production (i.e., CXCL8, IL1B, IL6 and TNFα) and (v) cell-mediated damage due to the increase in neutrophils number. In addition to cardiovascular damage, we observe skin alteration and inflammation. Finally, our data indicate a protective effect of HSA toward the toxins induced extra-intestinal effects. Together, our findings can serve as a starting point for humans’ studies to substantiate and understand the extra-intestinal effects observed in CDI patients.

## 1. Introduction

C. *difficile* is a gram-positive, anaerobic, spore-forming, toxin-producing bacillus, emerged as the main responsible for nosocomial infections in Western countries [1]. The clinical manifestations of *C. difficile* infection (CDI) range from asymptomatic colonization and mild diarrhea to toxic megacolon and life-threatening fulminant colitis [2]. The pathogenic effects of *C. difficile* are mainly caused by the production of toxins A (TcdA) and B (TcdB) in the host’s gut [3]. Both toxins require a receptor-mediated endocytosis to enter the cell and exert their cytotoxic effects [4]. Here, TcdA and TcdB monoglucosylate and inactivate the Rho GTPases expressed in the cytoplasm of host cells, causing cytopathic and cytotoxic effects and ultimately colonocytes death and loss of the intestinal barrier [5]. Beside antibiotics, the main risk factors for CDI infection include advanced age, impaired humoral immunity, renal disease and hypoalbuminemia [6]. In particular, an association between low human serum albumin (HSA) levels and CDI development, severity and mortality rates has been proposed in the last decade [7,8,9,10]. Part of this correlation seems to reside in HSA capability to bind and inactivate TcdA and TcdB, thus exerting a protective effect on *C. difficile*-induced host cell damage [11]. Unlike other bacteria, the septic process during CDI is not secondary to the bacterial bloodstream invasion but rather to the associated toxemia. Until recently, the phenomenon of extra-intestinal damage caused by *C. difficile* toxins has been largely undervalued. This is likely due to limits in detecting toxemia in humans, as the levels of circulating toxins are below the detection limit of existing assays [12]. Several studies have demonstrated that *C. difficile* could be responsible of systemic complications in human [13,14,15]. A systematic analysis of extra-intestinal CDIs observed during a 10-year study period was recently performed [16]. In this case, thirty-one of approximately eighteen thousand patients with *C. difficile* positive fecal samples developed extra-intestinal CDIs. The type of infection observed in these patients ranged from bacteremia, abdominal infection with or without surgery to perianal abscess and wound infection [16]. Authors concluded that extra-intestinal CDIs mainly occurred in hospitalized and antibiotic-treated patients who often had severe comorbidities. Extra-intestinal CDIs were mostly located in the abdominal area but the bacteremia associated with *C. difficile* could enter distant sites through transient bacteremia causing high mortality [16]. Another study found extra-intestinal CDIs in twenty-one of approximately two thousand and thirty-four patients with *C. difficile* positive isolation. Also in this case, most patients had comorbidities (diabetes, cancer, liver cirrhosis) and shown extra-intestinal involvement particularly in the abdominal area even if one patient developed brain abscesses [17].

Zebrafish embryo is a widely accepted model for the study of infectious diseases including CDI [11,18,19]. Zebrafish embryo provides several distinct advantages over the traditional animal models such as the transparent skin, which allows the direct visualization of toxin-induced changes in organs and physiology [20]. While TcdB has been reported to induce cardiotoxicity [18] and hemorrhagic effects on the vasculature system of zebrafish embryos [11], no data are available for TcdA systemic effects as well as with regard to a direct comparison between the different effects of TcdB and TcdA. Moreover, toxins effect on the immune system is still poorly understood. Thus, the aim of our study was to evaluate in vivo in a zebrafish model, the above aspects related to TcdA and TcdB intoxication. In addition, here we reinforce previous evidence [11] indicating the protective role of HSA towards toxins-induced extra-intestinal effects.

## 2. Materials and Methods

### 2.1. Zebrafish Care and Use Statement

The animal protocol was designed to minimize pain and discomfort to the embryos. All experimentations were conformed to the ITA guidelines (Dgl 26/2014) in accordance with EU legislation (2010/63/UE). The experimental protocol was approved by the Ethics Committee for animal experimentation (OPBA) of the University of Trieste (n. PO3118ZEN16). Adult fishes and embryos were maintained as previously described [11,20]. Tg(fli1:EGFP)y(1) zebrafish strain, that expresses in the entire vasculature EGFP under the control of the fli1 promoter [21], was kindly provided by Prof. Francesco Argenton (University of Padova, Padova, Italy). When needed, embryos were anesthetized by using 1:100 dilution of 4 mg/mL Tricane (Merck, Billerica, MA, USA) in experimental procedures.

### 2.2. Embryos Preparation and Treatment

Eight hours post fertilization (hpf), zebrafish embryos were treated with PTU (1-phenyl 2-thiourea, 0.03 mg/mL, Merck) to remain transparent for microscope analysis. At 24 hpf, zebrafish embryos were removed from the chorion and treated with HSA (0.5 mg/mL, Merck) and/or commercial *C. difficile* toxins TcdA (BML-G140-0050, batch #10051563, Enzo Life Sciences, Farmingdale, NY, USA) or TcdB (BML-G150-0050, batch #10051563, Enzo Life Sciences) as previously described [11]. Briefly, the embryos were exposed to a range of increasing doses for both toxins (from 1.5 µg/mL to 10 µg/mL and from 3 µg/mL to 10 µg/mL for TcdA and TcdB, respectively) to evaluate the sub-lethal concentrations, which were found to be 2.5 and 8 µg/mL for TcdA and TdB, respectively. Approximately 50 ng of HSA per embryo were directly injected into the cardiac venous sinus by using borosilicate glass micro-capillaries (20 μm O.D. 15 μm I.D.; Eppendorf AG, Hamburg, Germany) and a microinjector (FemtoJet^®^; Eppendorf AG). One hour after HSA-injection, TcdA or TcdB were directly added into the zebrafish embryos medium [18] at a final concentration of 2.5 µg/mL and 8 µg/mL, respectively. As control, zebrafish embryos were injected only with saline (vehicle, Braun AG, Melsungen, Germany), which was used to suspend the HSA powder. Embryos were then incubated for 24 h with toxins at 28 °C and repeatedly washed with E3 medium/PTU (1-phenyl 2-thiourea). To exclude any potential unspecific effect due to the contaminating lipopolysaccharide LPS in our toxins preparation, we treated embryos for 24 h with increasing doses of LPS produced by *E. coli* (L4524-5MG, Merck). Groups of at least 35 embryos for each condition were considered and each independent experiment was repeated thrice. Embryos were daily observed at the stereomicroscope (S8 APO, Leica Microsystems, Wetzlar, Germany) to evaluate vital signs (i.e., survival, heart rate) and any morphological defects (i.e., pericardial edema formation, length changing, bleeding). All pictures were taken at the same resolution and magnification with the live fishes positioned in 1.5% methylcellulose (Merck) in a lateral orientation and opportunely anesthetized.

### 2.3. Histology of the Skin

For the histologic analysis of the skin, embryos at 48 hpf (24 h after toxins treatment) were fixed in 4% buffered paraformaldehyde, dehydrated and paraffin-embedded. Thick sections (3 µm) were cut by Microm HM450 sliding microtome (Thermo Fisher Scientific, Waltham, MA, USA) and stained with hematoxylin (#H9627-25G, Merck) and eosin (#1159340025, Merck). Images of stained embryos were taken with a Leica DM-2000 microscope (Leica Microsystems).

### 2.4. Protein Extraction and Western Blot

Frozen larvae samples were manually homogenized in ice-cold RIPA Lysis Buffer 1X (Merck) added with protease and phosphate inhibitors (Merck). After an incubation of 15 min on ice, the homogenates were centrifuged at 13,000 rpm for 15 min at 4 °C and the supernatants of each sample were collected and store at −80 °C until protein quantification. Protein concentration was evaluated by BCA protein assay kit (Thermo Fisher Scientific) following the manufacturer’s instruction. Forty micrograms of whole protein extracts were loaded onto a 10% SDS-PAGE and blotted onto nitrocellulose filters (Merck). After blocking, membranes were incubated with primary antibody directed against ANP (#AB5490, rabbit, 1:800, Merck), BNP (sc-271185, 1:500, rabbit, Santa Cruz Biotechnology Inc., Dallas, TX, USA), followed by incubation with peroxidase-conjugated goat-anti mouse (sc-2005, Santa Cruz Biotechnology Inc., Dallas, TX, USA) or goat-anti rabbit (sc-2004, Santa Cruz Biotechnology Inc., Dallas, TX, USA) secondary antibodies. Immunoreactivity was detective using the Supersignal™ West Pico chemiluminescent substrate (Thermo Fisher Scientific). β-Actin (A3854-220UL, Merck) was used as loading control.

### 2.5. RNA Extraction and Real-Time Quantitative PCR (qRT-PCR)

Total RNA from at least 15 larvae for each experiment was isolated with Trizol^TM^ Reagent (Thermo Fisher Scientific) as previously described [22]. Gene expression was analyzed by Real-Time quantitative PCR (qRT-PCR) (Step One Plus, Applied Biosystems, Foster City, CA, USA) using the double strand DNA-binding dye SYBR Green (Thermo Fisher Scientific). Actin was used as the housekeeping gene. Primer sequences are listed in Supplementary Table S1.

### 2.6. Sudan Black Staining

Neutrophils were visualized by Sudan Black staining. Embryos were fixed at 48 hpf in 4% glutaraldehyde in phosphate-buffered saline PBS for 10 min, rinsed thrice in PBS, incubated in Triton 0.1%/PBS 5 min and then stained with 0.18% Sudan black (#BP109-10, Fisher Bio reagents) solution for 20 min. Embryos were washed extensively in 70% ethanol and then progressively rehydrated with PBS 1X containing increasing concentration of glycerol (30%, 50% and 80%; Merck). Images of stained embryos were taken with a Leica S8 APO stereomicroscope (Leica Microsystems).

### 2.7. Cell Culture Conditions

HUVEC (Human Umbilical Vein Endothelial Cells) cells were kindly provided by Dr. Fleur Bossi (IRCCS Burlo Garofolo, Trieste, Italy). Caco-2 were purchased from “Banca Biologica e Cell Factory—IST” (Istituto Nazionale per la Ricerca sul Cancro, Genova, Italy). HUVEC cells were cultured in complete F-12K medium supplemented with 20% newborn calf serum (Thermo Fisher Scientific), endothelial growth factor (50 mg/mL), heparin (50 mg/mL), penicillin (100 U/mL) and streptomycin (100 mg/mL) (Merck). Caco2 cells were grown in Dulbecco’s Modified Eagle Medium (DMEM) supplemented with 20% fetal bovine serum (FBS, Thermo Fisher Scientific), penicillin (100 U/mL) and streptomycin (100 mg/mL) (Merck). Cell lines were kept at 37 °C in a humidified atmosphere at 5% CO_2_.

### 2.8. MTT and LDH Assay

HUVEC and Caco2 cells were seeded at the density of 10^4^ or 3.4*10^4^ cells/cm^2^ in 96-wells plates, respectively. After 24 h, HUVEC cells were treated for further 24 h with increasing doses of TcdA (0.08, 0.157, 0.313, 0.625, 1.25, 2.5, 5 and 8 µg/mL) or TcdB (0.25, 0.5, 2, 4, 8 and 12 µg/mL). Caco2 monolayers were treated for 24 and 48 h by increasing doses of TcdA (0.02, 0.04, 0.08, 0.157, 0.313, 0.625, 1.25, 2.5, 5 and 7.5 µg/mL) and TcdB (0.0625, 0.125, 0.250, 0.5, 1, 2, 4 and 8 µg/mL). MTT (3-[4,5-dimethylthiazol-2-yl]-2,5 diphenyl tetrazolium bromide) assay was performed as previously described [23]. Briefly, MTT was given to cells at the final concentration of 0.5 mg/mL and incubated for 4 h at 37 °C. After that, cell medium was removed and salt crystals formed inside the cells solubilized with dimethyl sulfoxide (DMSO, Merck). Cellular cytotoxicity was evaluated by the Lactate Dehydrogenase (LDH) assay (Bio Vision Inc., Milpitas, CA, USA) on HUVEC cellular supernatant as previously reported [23]. Briefly, 100 μL of cellular supernatant were transferred to corresponding wells in an optical clear 96-well plate. 100 μL of reaction solution, consisting of catalyst solution mixed with dye solution (ratio 1:45), were then added to each well and incubated for 30 min at room temperature (RT), protecting the plate from light. Absorbance was measured at 500 nm using a spectrophotometer (Spectra Max Plus 384, Molecular Devices). As positive control, cells were treated with Triton X-100 (1% final concentration).

### 2.9. Immunostaining 

Cells were seeded at the density of 10^4^ cells/cm^2^ on glass slides located in 12-well plates. After 24 h, HUVEC cells were treated for further 24 h with either 2.5 µg/mL TcdA or 8 µg/mL TcdB, then fixed in 4% paraformaldehyde/0.15% picric acid for 20 min at room temperature (RT). After three washes with PBS 1X, cells were incubated with 0.1% Triton X-100 for 5 min, then with 0.5% BSA/PBS for 1 h and finally exposed to β-tubulin primary antibody (#T2200-200UL, rabbit, 1:200, Merck) overnight at 4 °C in a humid chamber. Cells were then incubated with the secondary anti-mouse Alexa Fluor 488 (#A11034, goat anti-rabbit, 1:400, Thermo Fisher Scientific) and phalloidin-TRITC dye (#P1951, Merck) for 1 h at RT in the dark. After washing, slides were mounted by Mowiol Mounting solution (Merck). Images were acquired with a Leica DM-2000 microscope (Leica Microsystems).

### 2.10. Image Analysis

All images were acquired with a Leica DM-2000 or a Leica S8AP0 stereo microscope. Heart chambers area, pericardial edema and the SIV vessel parameters (area, length and diameter) were measured by the ImageJ software using high-resolution images (40×). HUVEC cell rounding, expressed as the ratio of total and nuclear area, were also valuated by the ImageJ software (1.8.0 version).

### 2.11. Statistical Analysis

All experiments were performed at least thrice. Values, expressed as mean ± SEM, were analyzed using PRISM 6.0 (GraphPad Software, San Diego, CA, USA). ANOVA test was used to assess statistical significance. A *p* value < 0.05 was considered statistically significant.

### 2.12. Data Availability and Statement

Raw data and materials are available on request to corresponding author.

## 3. Results

### 3.1. Toxins Effects on Zebrafish Vitality

**Figure 1:**
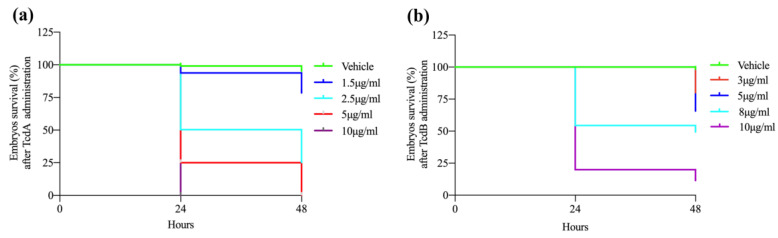
*C. difficile* toxins affect survival of zebrafish embryos. (**a**) Kaplan-Meier curve showing zebrafish embryo survival 24 and 48 h after treatment by increasing doses of TcdA (1.5, 2.5, 5 and 10 µg/mL). Data are presented as mean ± SEM. (*n* = 110 for each group) (*p* < 0.0001 by log-rank test). (**b**) Kaplan-Meier curve showing zebrafish embryo survival 24 and 48 h after treatment by increasing doses of TcdB (3, 5, 8 and 10 µg/mL). Data are presented as the mean ± SEM. (*n* = 110 for each group) (*p* < 0.0001 by log-rank test).

In order to establish the sub-lethal concentrations able to induce the toxins systemic effects, embryos were exposed to increasing doses of each toxin, ranging from 1.5 to 10 µg/mL for TcdA and from 3 to 10 µg/mL for TcdB (Figure 1a,b). We selected the sub-lethal doses of 2.5 µg/mL and 8 µg/mL for TcdA and TcdB respectively because, at these concentrations, toxins were able to induce morphological effects with a similar mortality rate compared to vehicle-treated embryos. At higher doses, both toxins showed a dramatic lethality rate (Figure 1a,b). In particular, Kaplan-Meier curves indicated a survival rate at 24 h post-treatment (hpt) of approximately 50% in embryos treated with 2.5 µg/mL and 8 µg/mL for TcdA and TcdB, respectively. The survival rate at 48 hpt decreased down to 25% in TcdA-treated animals and remained unchanged in TcdB-treated embryos (Figure 1a,b).

To support the concept that the above effects can be specifically ascribed to TcdA and TcdB, we confirmed the toxins activity on a control cell line (Caco2 cells) known to be susceptible to toxin effects (Supplementary Figure S1). Cell viability was assessed at both 24 and 48 hpt and showed a dose-dependent susceptibility of Caco2 cells to both *C. difficile* toxins, particularly after 48 h of treatment (Supplementary Figure S1a,b).

Moreover, to exclude any potential unspecific effect due to the contaminating LPS in our toxins preparation, we tested the doses 2.5 µg/mL and 8 µg/mL of LPS on 24-h embryos. Furthermore, we tested the dose of 70 µg/mL of LPS which, according to the literature, does not induce mortality in zebrafish embryos [24] but can produce visible toxic outcomes. The lack of any significant effects of LPS at the tested doses allowed us to exclude any unspecific LPS-related change (Supplementary Figure S2). To apply the same treatment protocol used for *C. difficile* toxins, LPS was added directly into the embryo’s growth medium. All the doses tested did not exert effects on the embryos survival (more than 90% of treated animals were alive compared to control), although embryos treated with 70 µg/mL of LPS showed a significant pericardial edema at 48 hpt compared to vehicle-treated animals (Supplementary Figure S2a,b). Furthermore, none of the tested doses increased the pro-inflammatory cytokines production (Supplementary Figure S2c). 

### 3.2. Toxins Effects on the Zebrafish Cardiac System

**Figure 2:**
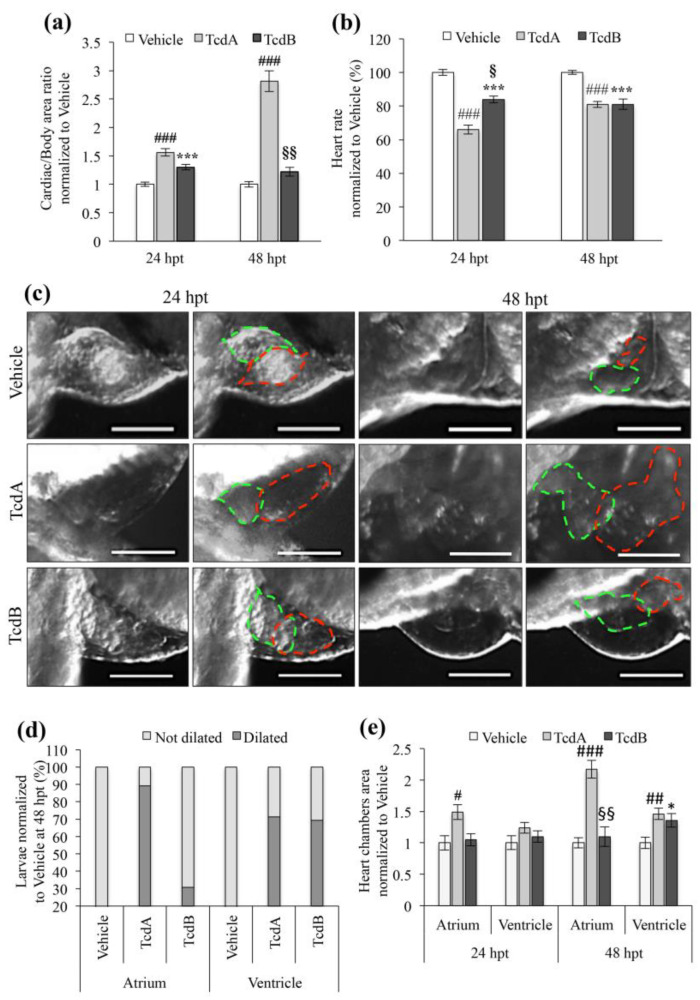
Effects of TcdA and TcdB treatment on zebrafish embryos heart. (**a**) Evaluation of pericardial edema in zebrafish embryos 24 and 48 h after administration of vehicle, 2.5 µg/mL TcdA or 8 µg/mL TcdB. The histogram displays the ratio of cardiac to total body area. Data are normalized to the vehicle group and are presented as the mean ± SEM. (*n* = 30 for each group), (^###^ *p* < 0.001, *** *p* < 0.001 compared to vehicle; ^§§^ *p* < 0.001 compared to TcdA). (**b**) Evaluation of the zebrafish embryo heart rate after toxins treatment (2.5 µg/mL TcdA or 8 µg/ mL TcdB). Data are presented as the mean ± SEM. (*n* = 50), (^###^ *p* < 0.001, *** *p* < 0.001 compared to vehicle; ^§^ *p* < 0.01 compared to TcdA). (**c**) Representative images of the embryos heart chambers after toxins (2.5 µg/mL TcdA or 8 µg/mL TcdB) or vehicle administration. On the left part on the panel, images taken at 24 hpt while on the right part of the panel images taken at 48 hpt. Red dashed lines show atrium while green one marked ventricle. Images were acquired with a Leica S8AP0 stereo microscope. Scale Bar = 200 µm. (**d**) Effects of 2.5 µg/mL TcdA or 8 µg/mL TcdB administration on the embryos heart chambers at 48 hpt. (*n* = 20). Data are normalized to vehicle and shown as the mean ± SEM (*n* = 20 for each group) (**e**) Quantization of embryos heart chamber area after toxins (2.5 µg/mL TcdA or 8 µg/mL TcdB) or vehicle treatment. Data, normalized to vehicle, are represented as the mean ± SEM. (*n* = 20 for each group), (^#^ *p* < 0.05, ^##^ *p* < 0.01, ^###^ *p* < 0.001, * *p* < 0.05 compared to vehicle; ^§§^ *p* < 0.001 compared to TcdA).

To assess the physiological changes in intoxicated embryos, we analyzed the presence of pericardial edema, a typical signal of pathological damage associated with cardiac and vascular defects and/or kidney failure in zebrafish [25,26,27]. At 24 hpt, both toxins caused a moderate pericardial edema that worsened to a significant severe edema condition in embryos treated with TcdA for 48 h. Pericardial edema remained unchanged in embryos following 48 h exposure to TcdB (Figure 2a, Figure 4a). 

Hamm et al. reported that TcdB acts on heart contractility by targeting directly cardiomyocytes [18]. First we analyzed the cardiac effects associated with either TcdA or TcdB administration, showing that the heart rate was significantly reduced after 24 and 48 h from embryos exposure to TcdA or TcdB (*p* < 0.001), compared to vehicle-treated animals (Figure 2b). Moreover, at 24 hpt the heart rate reduction was significantly higher in TcdA-treated embryos compared to TcdB-treated one (*p* < 0.01) (Figure 2b).

**Figure 3:**
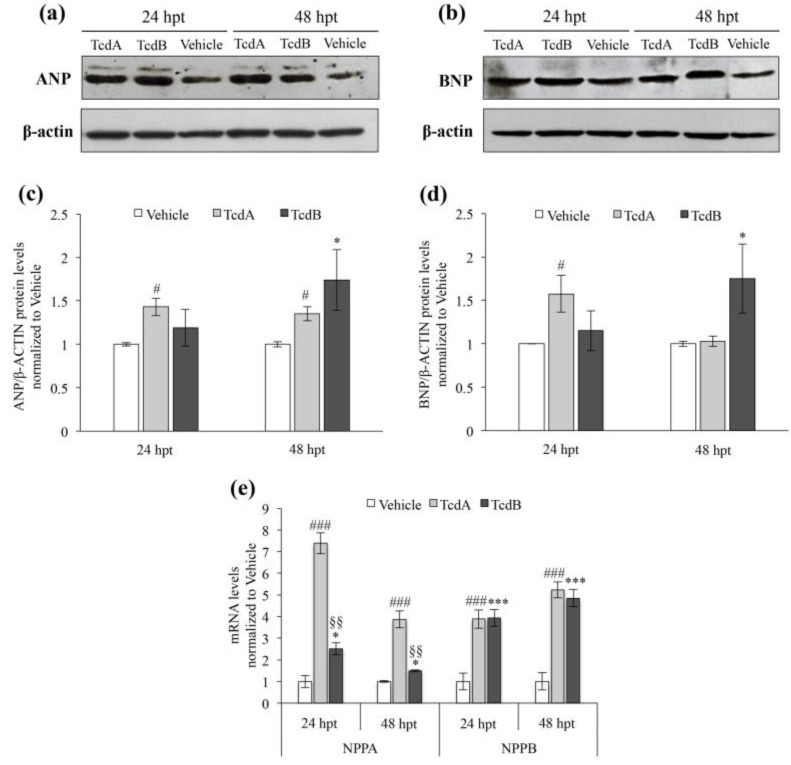
Cardiac hypertrophy markers analysis confirms the early role of TcdA in inducing heart damage compared to TcdB in zebrafish embryos. (**a**) Representative Western blot analysis for Atrial Natriuretic Factor (ANP) in vehicle, 2.5 µg/mL TcdA- or 8 µg/mL TcdB-treated embryos after 24 and 48 h. Actin is used to verify protein loading. (**b**) Representative Western blot analysis for Brain Natriuretic Peptide (BNP) in vehicle, 2.5 µg/mL TcdA- or 8 µg/mL TcdB-treated embryos at 24 and 48 h. Actin is used to verify protein loading. (**c**) Densitometric analysis of ANP protein in embryos treated with vehicle, 2.5 µg/mL TcdA or 8 µg/mL TcdB. Data, normalized to β-actin, are expressed as the mean ± SEM. (*n* = 5), (^#^ *p* < 0.05, * *p* < 0.05 compared to vehicle). (**d**) Densitometric analysis of BNP protein in embryos treated with vehicle, 2.5 µg/mL TcdA or 8 µg/mL TcdB. Data, normalized to β-actin, are expressed as the mean ± SEM. (*n* = 5), (^#^ *p* < 0.05, * *p* < 0.05 compared to vehicle). (**e**) qRT-PCR analysis of zebrafish NPPA (ANP) and NPPB (BNP) transcripts after 24 and 48 h of 2.5 µg/mL TcdA or 8 µg/mL TcdB treatment. Data, normalized to β-Actin mRNA level, are represented as the mean ± SEM. (*n* = 9), (^###^ *p* < 0.001, * *p* < 0.05 and *** *p* < 0.001 compared to vehicle; ^§§^ *p* < 0.001 compared to TcdA).

We also observed that the percentage of larvae with modified heart chambers areas showed an enlargement of the atrium in ~89% of TcdA-treated embryos (Figure 2d). The analysis of the heart chambers area highlighted by a significant enlargement of the atrium of TcdA-intoxicated animals at 24 hpt (*p* < 0.05), which worsened at 48 hpt (*p* < 0.001) in agreement with the formation of severe pericardial edema (Figure 2a,e). The ventricle of TcdA-treated embryos appeared enlarged only at 48 hpt compared to controls (*p* < 0.01; Figure 2e). On the contrary, TcdB did not exert any effect on the atrium, while an increase of the ventricle area was observed 48 hpt (*p* < 0.05; Figure 2e). Taken together these results suggest a more severe cardio-toxic effect of TcdA than TcdB. To investigate the molecular events underlying the cardio-toxic outcome of *C. difficile* toxins, we evaluated the expression of some hormones released by the heart chambers in response to hypertrophic stimuli (Figure 3).

The levels of the atrial natriuretic peptide (ANP) and brain natriuretic peptide (BNP), which are secreted from cardiomyocytes into the bloodstream in response to myocardial stretching [28], were analyzed (Figure 3a,b). Our data showed that ANP and BNP protein levels were already increased in TcdA-treated embryos at 24 hpt (1.4-fold and 1.6-fold, respectively, compared to vehicle-treated animals; *p* < 0.05; Figure 3c,d). At 48 hpt, ANP protein level remained unchanged in TcdA-treated embryos (1.3-fold compared to vehicle; *p* < 0.05; Figure 3c) while the amount of BNP protein returned to normal level (Figure 3d). On the contrary, in TcdB-treated animals a significant increase of ANP and BNP was observed only at 48 h (1.7-fold and 1.8-fold, respectively, compared to vehicle-treated animals; *p* < 0.05; Figure 3c,d). This behavior was comparable to that observed when evaluating the mRNA expression of both hypertrophy markers (Figure 3e).

### 3.3. Toxins Effects on the Zebrafish Vascular System 

**Figure 4:**
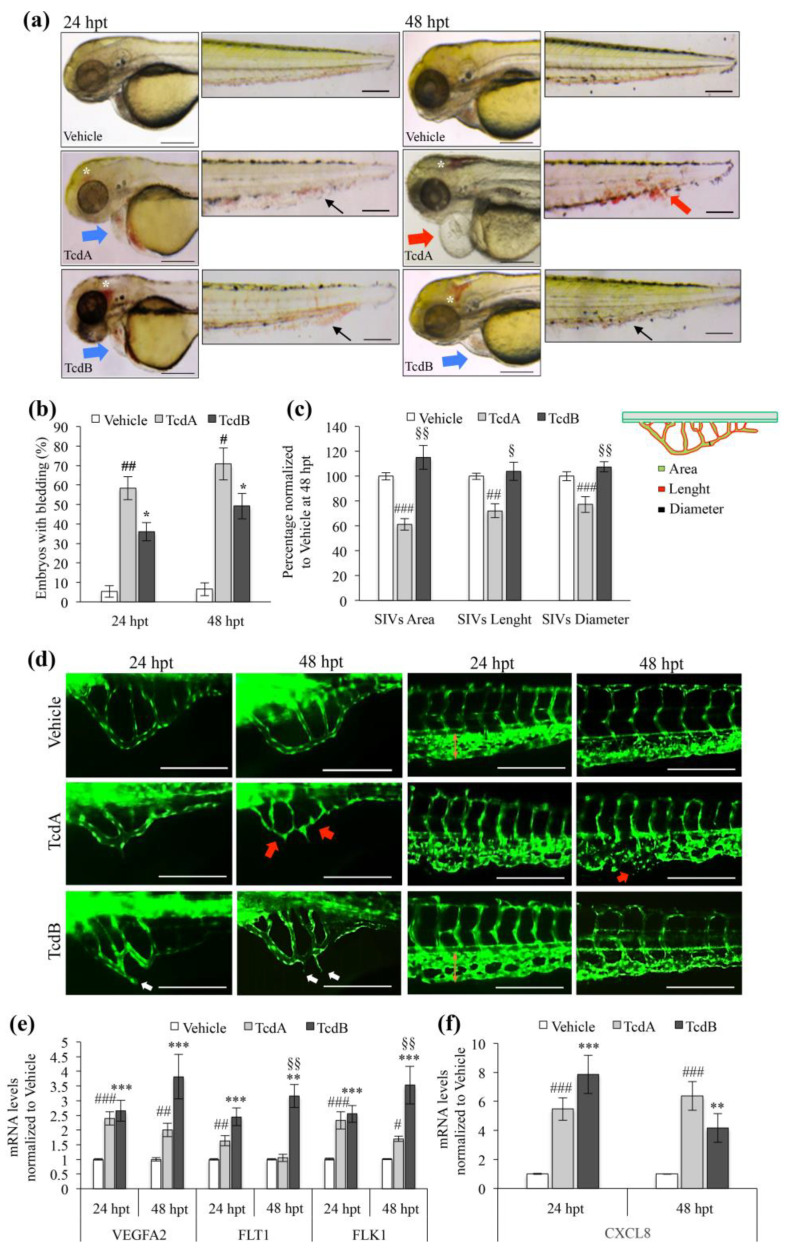
TcdA and TcdB induce different effects on the vascular system of zebrafish embryos. (**a**) Representative images of zebrafish embryos exposed to vehicle, 2.5 µg/mL TcdA or 8 µg/mL TcdB for 24 or 48 h. Vehicle-, TcdA- and TcdB-treated embryos are shown in the upper, middle and bottom panel, respectively. White asterisks indicate cerebral bleeding whereas black and red arrows show severe vascular damage observed at the tail level. Blue or red arrows indicate moderate or severe pericardial edema, respectively. Images were acquired with a Leica S8 AP0 stereo microscope. Scale bar = 200 µm. (**b**) The histogram showing the percentage of zebrafish embryos that exhibit bleeding after 2.5 µg/mL TcdA or 8 µg/mL TcdB addition. Data are presented as the mean ± SEM. (*n* = 6), (^#^ *p* < 0.05, ^##^ *p* < 0.01, * *p* < 0.05 compared to vehicle). (**c**) The area, length and diameter of the sub-intestinal veins (SIVs) are measured in zebrafish embryos 48 h after 2.5 µg/mL TcdA or 8 µg/mL TcdB administration. A schematic representation of the vessels is reported on the top of the graph and colored in green, red and black to indicate area, length and diameter, respectively. Data, normalized to vehicle, are represented as the mean ± SEM. (*n*  =  25), (^##^ *p* < 0.01, ^###^ *p* < 0.001, compared to vehicle; ^§^ *p* < 0.01 ^§§^ *p* < 0.001 compared to TcdA). (**d**) Representative images of sub-intestinal veins (SIVs; left panel) and tail vessels (right panel) taken at 24 or 48 h after toxins (2.5 µg/mL TcdA or 8 µg/mL TcdB) or vehicle administration on Tg(fli1:EGFP)Y1 strain embryos. Red arrows show ischemic processes of the sub-intestinal veins SIVs and tail whereas white arrows indicate ectopic processes sprouted from SIVs. Images were acquired with a Leica DM-2000 microscope. Scale bar = 200 µm. (**e**) qRT-PCR analysis of zebrafish VEGFA2, FLT1 and FLK1 after 2.5 µg/mL TcdA or 8 µg/mL TcdB treatment. Data, normalized to the zebrafish β-Actin mRNA amount, and represented as the mean ± SEM. (*n* = 9), (^###^ *p* < 0.001, ^##^ *p* < 0.01, ^#^ *p* < 0.05, *** *p* < 0.001 and ** *p* < 0.01 compared to vehicle; ^§§^ *p* < 0.001 compared to TcdA). (**f**) qRT-PCR analysis of zebrafish CXCL8 (IL-8) after 2.5 µg/mL TcdA or 8 µg/mL TcdB administration. Data, normalized to the β-Actin mRNA amount, are represented as the mean ± SEM. (*n* = 9), (^###^ *p* < 0.001, *** *p* < 0.001 and ** *p* < 0.01 compared to vehicle).

The most evident alteration to the vascular system driven by *C. difficile* toxins was the presence of bleeding sites in the head, particularly in the midbrain (white asterisk in Figure 4a) of embryos treated with either TcdA or TcdB. In addition, most of the animals treated with TcdA were characterized by a severe bleeding phenotype at the tail level (Figure 4a, middle panel, black and red arrows; Figure 4b). Indeed, the percentage of embryos with bleeding increased up to 58% in TcdA- (*p* < 0.01) and 36% in TcdB- (*p* < 0.05) treated animals (Figure 4b) at 24 hpt. At 48 hpt, the bleeding rate further rose to 70% in TcdA-intoxicated embryos, (*p* < 0.05) and 50% in TcdB-treated embryos (*p* < 0.05.), compared to vehicle (Figure 4b). Overall, TcdB-treated animals showed a lower rate of blood accumulation out the vascular system compared to TcdA-intoxicated animals (Figure 4a,b).

We further characterized the vascular effects induced by toxins focusing on the structural changes of the sub-intestinal veins (SIVs) and on the vascularization of the tail in vivo (Figure 4d). This analysis was conducted with animal expressing the enhanced green fluorescent protein EGFP in the endothelial cells to precisely detecting vessel morphology. The supra-intestinal artery (SIA) and sub-intestinal vein (SIV), the vessels system located into the yolk, compose the intestinal vasculature in zebrafish. Alterations in this structure could be associated with defective intestinal development and consequent dysfunctions [29]. Both toxins were able to completely subvert SIVs structure (Figure 4d, left panel). In particular, in TcdA-treated embryos the SIVs area was markedly decreased mainly at 48 hpt compared to vehicle-treated animals with a reduction in both vessel diameter and length (Figure 4c,d, red arrows). In contrast, embryos treated for 24 and 48 h with TcdB showed many ectopic processes sprouted from SIVs (Figure 4d, white arrows), which suggests proangiogenic alterations. Moreover, we observed an increase in the distance between the dorsal aorta (DA) and the posterior caudal vein (PCV) and in the thickness of the tail vessels, particularly marked at 24 hpt (Figure 4d, bottom right panel). These data suggest the proangiogenic stimulus induced by TcdB. On the contrary, TcdA promoted loss of tail vessels structure particularly 48 h after treatment (Figure 4d, middle right panel, red arrow). To confirm the above observations at the molecular level, we evaluated the transcript abundance of some angiogenic markers, such as the vascular endothelial growth factor (VEGFA2), a well-known regulator of vascular remodeling, and its two receptors (FLT1 and FLK1) (Figure 4e). The mRNA levels of VEGFA2 and of FLT1/FLK1 significantly increased in intoxicated embryos at both 24 and 48 hpt, although the phenomenon was less evident in TcdA-treated embryos than in TcdB-treated animals, in particular at 48 hpt (Figure 4e). Since it is known that VEGF-A promotes the production of pro-inflammatory cytokines, we also analyzed the transcripts levels of CXCL8, which usually correlates with high VEGF-A levels in human colonocytes treated with TcdA [30]. A significant increase of CXCL8 mRNA levels was observed after embryos treatment by either TcdA (*p* < 0.001 at 24 and 48 hpt) or TcdB (*p* < 0.001 at 24 and *p* < 0.01 at 48 hpt) (Figure 4f). 

**Figure 5:**
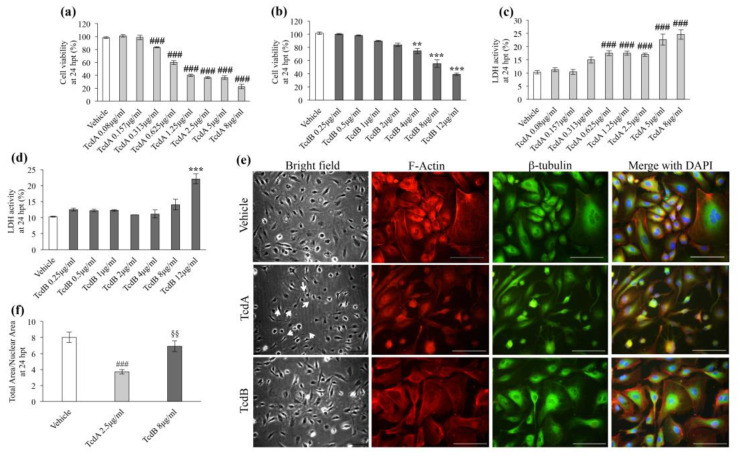
*C. difficile* toxins induce different effects on the morphology of human umbilical vein endothelium cells (HUVEC). (**a**) Cell viability, evaluated by MTT assay, in HUVEC cells treated with vehicle and increasing doses of TcdA. Data, normalized to vehicle, are represented as the mean ± SEM. (*n* = 6), (^###^ *p* < 0.001 compared to vehicle. (**b**) Cell viability, evaluated by MTT assay, in HUVEC cells treated with vehicle and increasing doses of TcdB. Data, normalized to vehicle, are represented as the mean ± SEM. (*n* = 6), (*** *p* < 0.001, ** *p* < 0.01 compared to vehicle. (**c**) Analysis of lactate dehydrogenase LDH activity on the supernatant of TcdA-treated HUVEC cells. Data, normalized to Triton X-100-treated cells, are represented as the mean ± SEM (*n* = 6), (^###^ *p* < 0.001 compared to vehicle). (**d**) Analysis of lactate dehydrogenase (LDH) activity on the supernatant of TcdB-treated HUVEC cells. Data, normalized to Triton X-100-treated cells, are represented as the mean ± SEM (*n* = 6), (*** *p* < 0.001 compared to vehicle). (**e**) Representative images of HUVEC treated with vehicle, 2.5 μg/mL TcdA or 8 μg/mL TcdB for 24 h. Images of cellular morphology are taken in bright field (left) Scale bar = 50 µm. In the middle of the panel, images in red and green represent the F-actin and the β-tubulin staining, respectively. On the right, the merge of the red and green fields with DAPI (blue) was shown. Images were acquired with a Leica DM-2000 microscope. Scale Bar = 50 µm. (**f**) HUVEC cells rounding, expressed as the ratio of total to nuclear area, 24 h after addition of vehicle, 2.5 μg/mL TcdA and 8 μg/mL TcdB. Data are represented as the mean ± SEM (*n* = 20), (^###^ *p* < 0.001 compared to vehicle, ^§§^ *p* < 0.01 compared to 2.5 μg/mL TcdA).

To determine the relevance of the effects observed in zebrafish in human cells, we studied the effects of toxins on human umbilical vein endothelium cells (HUVEC) (Figure 5).

The effects of increasing doses of TcdA or TcdB on cell viability were tested by MTT and LDH assays. While TcdA affected cells vitality even at low doses (from 0.313 μg/mL to 8 μg/mL; ^###^ *p* < 0.001) (Figure 5a), TcdB induced significant cell mortality at higher doses than TcdA, starting from 4 μg/mL (** *p* < 0.01 and *** *p* < 0.001) (Figure 5b). Consistent with MTT data, LDH test showed that a wide range of TcdA doses exerted significant cytotoxic effects compared to vehicle-treated cells (from 0.313 μg/mL to 8 μg/mL; ^###^ *p* < 0.001) (Figure 5c). In contrast, TcdB induced a significant cytotoxicity only at the dose of 12 µg/mL (*** *p* < 0.001) (Figure 5d). Further experiments, aimed at evaluating the morphological effects of *C. difficile* toxins on HUVEC cells, were carried out by choosing the doses used for the in vivo experiments, that is, 2.5 µg/mL or 8 µg/mL for TcdA or TcdB, respectively. The treatment with 2.5 µg/mL TcdA for 24 h destroyed HUVEC cells monolayer causing a significant cells rounding (Figure 5e,f, bright field). Immunofluorescence staining of F-actin and β-tubulin showed a severe thickening of F-actin stress fibers and a significant rearrangement of the cytoskeletal structure (Figure 5e). On the contrary, fewer morphological changes were observed in cells treated with 8 µg/mL TcdB compared to TcdA-treated cells even though cells showed a mild perinuclear thickening of the β-tubulin fibers (Figure 5e). These results support the different impact of TcdA and TcdB observed in the vascular structure of zebrafish embryos.

### 3.4. C. difficile Toxins Activate Zebrafish Immune System and Inflammation

**Figure 6:**
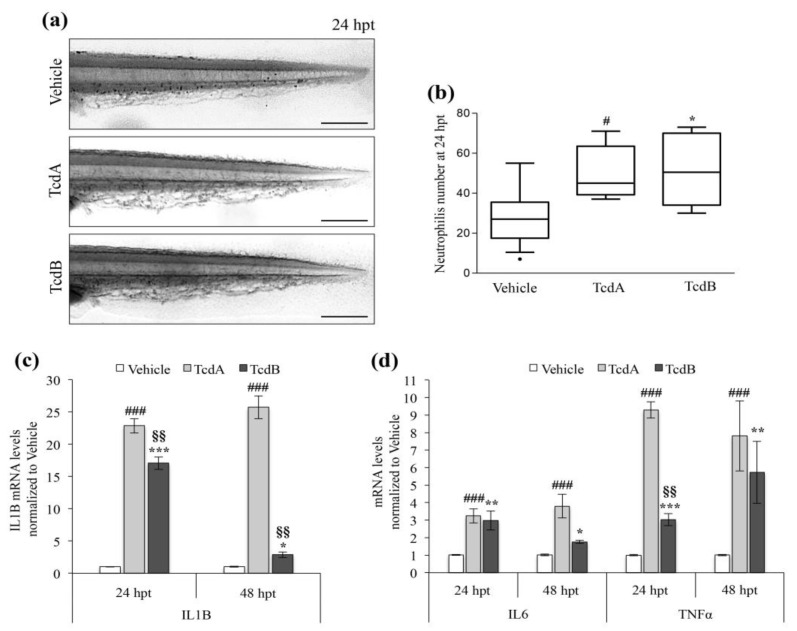
TcdA and TcdB promote the inflammatory response and active the immune system. (**a**) Representative images of Sudan Black staining of neutrophils at 24 hpt in zebrafish embryos treated with vehicle, 2.5 µg/mL TcdA or 8 µg/mL TcdB. The pictures are lateral view of the tail. Images were acquired with a Leica S8AP0 stereo microscope. Scale bar = 200 µm. (**b**) Neutrophils number in embryos treated with toxins (2.5 µg/mL TcdA or 8 µg/mL TcdB) or vehicle. Data are represented in a Tukey box and whisker plot, (*n* = 20), (^#^ *p* < 0.05, * *p* < 0.05 compared to vehicle). (**c**) qRT-PCR analysis of zebrafish pro-inflammatory cytokine IL1B at 24 and 48 h after the administration of 2.5 µg/mL TcdA or 8 µg/mL TcdB. Data, normalized to the β-Actin mRNA amount, are represented as the mean ± SEM. (*n* = 9), (^###^ *p* < 0.001, *** *p* < 0.001 and * *p* < 0.05 compared to vehicle; ^§§^ *p* < 0.001 compared to TcdA). (**d**) qRT-PCR analysis of zebrafish pro-inflammatory cytokines IL6 and TNFα at 24 and 48 h after the administration of 2.5 µg/mL TcdA or 8 µg/mL TcdB. Data, normalized to the β-Actin mRNA amount, are represented as the mean ± SEM (*n* = 9), (^###^ *p* < 0.001, *** *p* < 0.001, ** *p* < 0.01, * *p* < 0.05 compared to vehicle; ^§§^ *p* < 0.001 compared to TcdA).

Human CDI is characterized by neutrophil recruitment and acute and intense inflammatory response with secretion of pro-inflammatory cytokines [31]. At 24 hpt, we observed a 1.6 ~ fold increase of neutrophils number in the tail of TcdA- (*p* < 0.05) or TcdB- (*p* < 0.05) treated embryos, compared to vehicle-treated animals (Figure 6a,b).

Similar to what is described in humans [31], zebrafish intoxication by *C. difficile* toxins caused a significant increase in IL1B transcript at 24 hpt in TcdA- or TcdB-treated embryos compared to controls (~22-fold (*p* < 0.001) and ~17-fold (*p* < 0.001) respectively) (Figure 6c). At 48 hpt, the transcript levels of IL1B remained significantly elevated (~25-fold, *p* < 0.001) in TcdA-treated embryos; the amount of IL1B mRNA was only slightly augmented in animals incubated by TcdB compared to the control (Figure 6c). Finally, an increased expression of the pro-inflammatory cytokines IL6 and TNF-α was reported in both TcdA- or TcdB- treated embryos at 24 and 48 hpt compared to vehicle-treated animals (Figure 6d). Overall, the results obtained suggest that TcdA induced a stronger inflammatory response compared to TcdB. 

### 3.5. TcdA Induces Skin Alteration in Zebrafish Embryos

**Figure 7:**
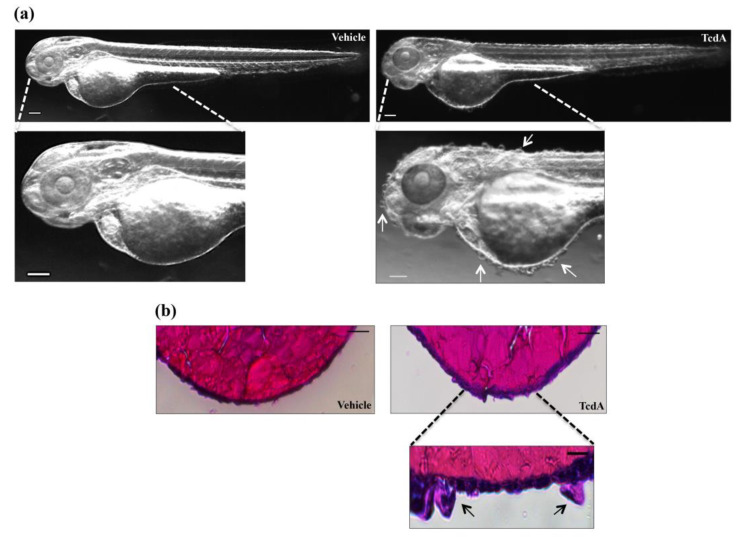
TcdA administration induced skin alteration in zebrafish embryos. (**a**) Representative images of 2.5 µg/mL TcdA- or vehicle-treated embryos. Images were acquired with a Leica S8AP0 stereo microscope. Scale bar = 100 µm. (**b**) Hematoxylin and eosin staining is performed on 2.5 µg/mL TcdA- or vehicle-treated embryos to evaluate skin structure. Scale bar = 100 µm (upper panel). A magnification of the hypertrophic cell of the epidemical layer is taken from TcdA-treated embryo (lower panel; scale bar = 10 µm). Images were acquired with a Leica S8AP0 stereo microscope.

A peculiar feature associated only with TcdA treatment was a significant alteration of the skin that appeared bullous throughout the body (Figure 7a, right panel, white arrows). Histological analysis of TcdA-treated embryos showed thickened skin and hypertrophy of some cells of the outermost layer of the epidermis shown by arrows in Figure 7b (lower-right panel). In contrast, no changes in the skin structure of TcdB-treated embryos were observed.

### 3.6. Human Serum Albumin Protects Zebrafish Embryos towards TcdA Intoxication

**Figure 8:**
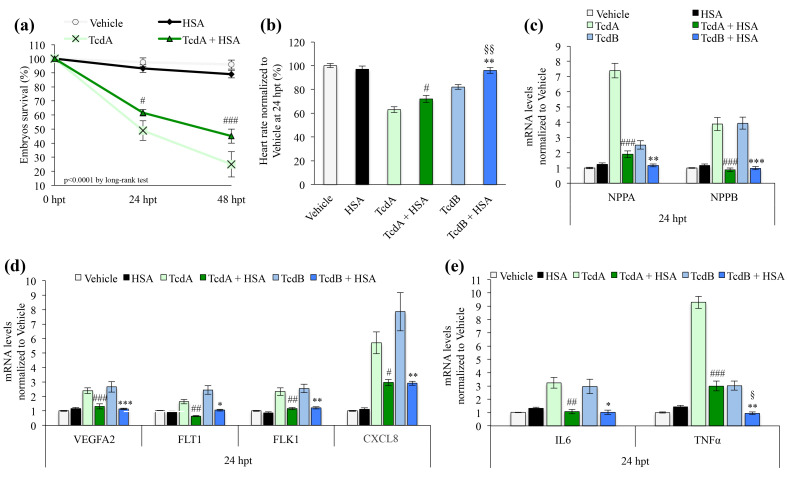
Human serum albumin (HSA) protects zebrafish embryos towards *C. difficile* toxins intoxication. (**a**) Kaplan-Meier curve showing zebrafish embryo survival 24 and 48 h after treatment with vehicle, 50 ng HSA, 2.5 µg/mL TcdA or TcdA+HSA. Data are presented as the mean ± SEM. (*n* = 110 for each group) (*p* < 0.0001 by log-rank test; ^#^ *p* < 0.05 TcdA compared to TcdA+HSA at 24 hpt; ^###^ *p* < 0.001 TcdA compared to TcdA+HSA at 48 ht). (**b**) Evaluation of the zebrafish embryo heart rate after treatment with vehicle or toxins (2.5 µg/mL TcdA or 8 µg/mL TcdB) and/or HSA. Data are presented as the mean ± SEM. (*n* = 40) (^#^ *p* < 0.05 compared to TcdA, ** *p* < 0.01 compared to TcdB; ^§§^ *p* < 0.001 compared to TcdA+HSA). (**c**) qRT-PCR analysis of zebrafish NPPA and NPPB after 24 h of 2.5 µg/mL TcdA+HSA or 8 µg/mL TcdB-+HSA co-treatments. Data, normalized to the zebrafish β-Actin mRNA level, are compared to vehicle and represented as the mean ± SEM (*n* = 9) (^###^ *p* < 0.001 compared to TcdA, ** *p* < 0.01 and *** *p* < 0.001 compared to TcdB). (**d**) qRT-PCR analysis of zebrafish VEGFA2, FLT1, FLK1 and CXCL8 (IL-8) after 24 h of co-treatment with either 2.5 µg/mL TcdA+HSA or 8 µg/mL TcdB+HSA. Data, normalized to the zebrafish β-Actin mRNA amount, are compared to the vehicle and represented as the mean ± SEM (*n* = 9) (^#^ *p* < 0.05, ^##^ *p* < 0.01 and ^###^ *p* < 0.001 compared to TcdA; * *p* < 0.05, ** *p* < 0.01 and *** *p* < 0.001 compared to TcdB). (**e**) qRT-PCR analysis of zebrafish IL6 and TNFα after 24 h of co-treatment with either 2.5 µg/mL TcdA+HSA or 8 µg/mL TcdB+HSA. Data, normalized to the β-Actin mRNA level, are compared to vehicle and represented as the mean ± SEM (*n* = 9) (^###^ *p* < 0.001 and ^##^ *p* < 0.01 compared to TcdA; ** *p* < 0.01 and * *p* < 0.05 compared to TcdB; ^§^ *p* < 0.01 compared to TcdA+HSA). Human serum albumin (HSA) protects zebrafish embryos towards *C. difficile* toxins intoxication. (**a**) Kaplan-Meier curve showing zebrafish embryo survival 24 and 48 h after treatment with vehicle, 50 ng HSA, 2.5 µg/mL TcdA or TcdA+HSA. Data are presented as the mean ± SEM. (*n* = 110 for each group) (*p* < 0.0001 by log-rank test; ^#^ *p* < 0.05 TcdA compared to TcdA+HSA at 24 hpt; ^###^ *p* < 0.001 TcdA compared to TcdA+HSA at 48 ht). (**b**) Evaluation of the zebrafish embryo heart rate after treatment with vehicle or toxins (2.5 µg/mL TcdA or 8 µg/mL TcdB) and/or HSA. Data are presented as the mean ± SEM. (*n* = 40) (^#^ *p* < 0.05 compared to TcdA, ** *p* < 0.01 compared to TcdB; ^§§^ *p* < 0.001 compared to TcdA+HSA). (**c**) qRT-PCR analysis of zebrafish NPPA and NPPB after 24 h of 2.5 µg/mL TcdA+HSA or 8 µg/mL TcdB-+HSA co-treatments. Data, normalized to the zebrafish β-Actin mRNA level, are compared to vehicle and represented as the mean ± SEM (*n* = 9) (^###^ *p* < 0.001 compared to TcdA, ** *p* < 0.01 and *** *p* < 0.001 compared to TcdB). (**d**) qRT-PCR analysis of zebrafish VEGFA2, FLT1, FLK1 and CXCL8 (IL-8) after 24 h of co-treatment with either 2.5 µg/mL TcdA+HSA or 8 µg/mL TcdB+HSA. Data, normalized to the zebrafish β-Actin mRNA amount, are compared to the vehicle and represented as the mean ± SEM (*n* = 9) (^#^ *p* < 0.05, ^##^ *p* < 0.01 and ^###^ *p* < 0.001 compared to TcdA; * *p* < 0.05, ** *p* < 0.01 and *** *p* < 0.001 compared to TcdB). (**e**) qRT-PCR analysis of zebrafish IL6 and TNFα after 24 h of co-treatment with either 2.5 µg/mL TcdA+HSA or 8 µg/mL TcdB+HSA. Data, normalized to the β-Actin mRNA level, are compared to vehicle and represented as the mean ± SEM (*n* = 9) (^###^ *p* < 0.001 and ^##^ *p* < 0.01 compared to TcdA; ** *p* < 0.01 and * *p* < 0.05 compared to TcdB; ^§^ *p* < 0.01 compared to TcdA+HSA).

We have previously demonstrated that the injection of HSA in the vascular system of zebrafish embryos attenuates the in vivo effects associated with TcdB exposure [11]. In particular, we reported a higher survival of HSA-TcdB co-treated embryos compared to TcdB-treated embryos, together with an amelioration of the heart rate. Moreover, in co-treated animals we observed a reduction of the pericardial edema compared to TcdB-treated embryos at 24 hpt. Here, we explored the potential protective role of HSA towards TcdA intoxication in comparison to the protective effects of HSA towards TcdB (Figure 8).

We observed that HSA increased the survival of embryos exposed to TcdA (*p* < 0.05 TcdA vs. TcdA+HSA at 24 hpt; *p* < 0.001 TcdA vs. TcdA+HSA at 48 ht; Figure 8a). Moreover, the heart rate significantly increased in embryos pre-treated with HSA before intoxication with TcdA compared to animal exposed to TcdA alone (*p* < 0.05; Figure 8b). However, the protective effect of HSA towards TcdA appears to be less pronounced compared to that reported for TcdB (*p* < 0.01; Figure 8b) [11]. The analysis of the hypertrophy markers ANP (NPPA) and BNP (NPPB) showed that the incubation with HSA before toxins administration was able to protect the heart against hypertrophy mechanisms (Figure 8c). In particular, HSA pre-treatment before TcdA intoxication caused a 3.8- and 4.4-fold decrease of NPPA and NPPB mRNA levels, respectively, compared to TcdA-treated embryos (Figure 8c). Moreover, HSA pre-treatment before TcdB intoxication caused a 2.2- and 4-fold decrease of NPPA and NPPB mRNA levels, respectively, compared to TcdB-treated embryos (Figure 8c). 

The evaluation of proangiogenic markers showed that HSA restores the levels of VEGFA2, FLT1 and FLK1 in toxin-treated embryos to values comparable to vehicle-treated embryos, thus demonstrating a complete inhibition of the toxins activity (Figure 8d). In addition, the reduction of the pro-inflammatory cytokine CXCL8, induced by VEGFA (Figure 8d) confirmed the protective effect of HSA. 

Finally, with regard to the effects on the immune system, qRT-PCR analysis showed a statistically significant decrease of the mRNA levels of both IL6 and TNF-α in embryos exposed to HSA before intoxication with either TcdA or TcdB (Figure 8e).

## 4. Discussion

CDI is not only a “gut-confined” disease as it is possible that the bloodstream invasion by toxins could be the key to determine the systemic effects seen in some CDI patients. Moreover, although the infection mainly affects frail older or hospitalized and antibiotic-treated patients, who often had severe comorbidities, CDI-associated mortality is not merely explained by diarrhea and dehydration. Despite extra-intestinal CDIs are rare considering the high number of hospitalized patients with *C. difficile* positive fecal samples, many studies reported that *C. difficile* could be responsible of systemic complications in human [13,14,15]. The systemic effects could range from bacteremia, abdominal infection with or without surgery to perianal abscess and wound infection [16,17]. It should be also considered that the number of patients with extra-intestinal CDIs could be under-estimated as *C. difficile* might be difficult to isolate and the rate of recovery might vary depending on the isolation technique [16,32]. In addition, *C. difficile* was often isolated with other microbes, which could make its identification more difficult. 

From clinical practices, we have noticed a relative bradycardia in human patients with severe CDI (normal heart rate despite fever and significant water losses) and this observation have contributed to drive our investigations. Based on these clinical observations, we have investigated the extra-intestinal effects of *C. difficile* toxins taking into account that other members of the phylum *Firmicutes* (e.g., *C. perfringens*, *C. botulinum*, *C. tetani*, *C. diphtheriae*, *B. anthracis,* *S. pneumoniae* and *S. pyogenes*) are toxicogenic Gram positive (most of them spore-forming) with peculiar cardiac and neuronal toxicity potential [33]. Indeed, in vivo models and clinical data indicate that: (i) *C. perfringens* toxins cause reduced cardiac output, hypotension and bradycardia in rabbits [34]; (ii) the most common complication in patients with *C. diphtheriae* infection is myocarditis [35]; (iii) patients surviving beyond the acute phase of *C. tetani* infection show dysrhythmias and myocardial infarction [36] (iv) *B. anthracis* lethal toxin-induced cardiotoxicity has been proposed [37] and (v) exotoxins produced by *S. pneumoniae* and *S. pyogenes* have been described to cause myocardial and endothelial dysfunctions, both in vitro and in clinical studies [38,39,40]. Although a recognized cardiovascular pathogenicity has never been associated to CDI, in vivo zebrafish embryo models indicated the cardio-tropism and cardiotoxicity of *C. difficile* toxins [11,18].

Here, we demonstrate that both TcdA and TcdB toxins produced by *C. difficile* have a cardio-toxic potential (Figure 2 and Figure 3). The cardiac damage has been highlighted by several markers: inflammation (i.e., pericardial edema, Figure 2a), “defense mechanism” (i.e., cardiac hypertrophy) (Figure 2c–e), rhythm disturbances with likely conduction system damage (i.e., decrease in heart rate) (Figure 2b), hormonal reaction (i.e., increase of ANP and BNP) (Figure 3) and vascular endothelium damage (Figure 4). Altogether, these findings are suggestive for a multi-target cardiac damage resembling those reported in myocarditis [41]. Despite this, the mechanisms of action of TcdA and TcdB seems to be somehow different. Indeed, although both toxins stimulate the release of hormones involved in hypertrophic mechanisms (Figure 3), TcdA-induced damage seems primarily to affect the atrial structure and then extend to ventricle while TcdB appears to act mainly at the ventricular level (Figure 2c–e). Moreover, the cardio-toxic action of TcdA appears earlier than that of TcdB, which, in contrast, induces significant effects only after 48 h of incubation (Figure 2c–e; Figure 3c,d).

*C. difficile* toxins activity also affects the vessels system. As with cardiac damage, the vascular injury caused by the two toxins appears to be very different (Figure 4a,d). Indeed, TcdA seems to have an anti-angiogenic effect on SIVs vessels and a high cytotoxic outcome (Figure 4c,d), as also supported by results obtained in HUVEC cells (Figure 5). On the contrary, TcdB shows milder effects based on the promotion of ectopic vessel sprouting (Figure 4d, left-bottom panel, white arrows). In line with the literature [30], here we show that both toxins were able to increase the mRNA levels of VEGFA2 as well as of its receptors, FLT1 and FLK1 (Figure 4e). In fact, it is already known that VEGFA/FLK1 signaling is necessary for early intestinal vasculature development by directing the migration and survival of endothelial cells [29]. Furthermore, VEGFA and its receptors are involved not only in the proangiogenic mechanisms but also in the endothelial response to oxygen reduction, which typically occurred in the ischemic process [42]. Thus, it is possible that the toxin-induced increase in the VEGFA mRNA levels reflects the activation of vascular remodeling driven by pro-angiogenetic (TcdB) or ischemic (TcdA) stimuli.

Hamm and coworkers have previously demonstrated that TcdB exhibits heart-specific tropism, showing that TcdB can be detected in both cardiac and SIVs regions 24 h after toxin administration [18]. Our results, showing cardiac morphological changes, further support this evidence. Furthermore, since the activation of the cardiac endothelium and the overlying epicardium has been described as one of the earliest responses to cardiac injury in zebrafish [43], it is possible that these mechanisms occur simultaneously.

A toxin-mediated increase in hemorrhagic sites and embryos bleeding (Figure 4a,b), as well as an increased neutrophils number (Figure 6a,b), has been observed in intoxicated embryos. Zebrafish neutrophils are fully functional in the very early developmental stages and capable of phagocytosis by 28–30 hpf [44]. As in human, various inflammatory mediators, including IL8 (CXCL8), drive neutrophils recruitment and recent studies reveal that neutrophils are also involved in the production of chemokines in response to a variety of stimulants including TNFα. Moreover, activated neutrophils exhibit many activities including angiogenesis through the production of VEGF [45]. Based on these evidences, increased levels of pro-inflammatory cytokines (i.e., CXCL8) (Figure 4f) in toxin-treated embryos could promote chemotaxis, induce hematopoiesis through an increase in neutrophils number and induce vascular permeability in a VEGF-dependent way. Moreover, the significantly increase of the transcript levels of IL1β and IL6 mRNA in zebrafish embryos treated with both toxins (Figure 6c,d) reflects a wide toxins-related pro-inflammatory cascade such as in human [46].

A peculiar morphological effect associated only with TcdA treatment is the presence of bubbles on the embryo’s skin surface (Figure 7a, lower-right panel, white arrows). Moreover, the hematoxylin and eosin staining shows that the outermost layer of the epidermis in TcdA-treated embryos appeared thickened compared to vehicle-treated ones (Figure 7b, lower-right panel, black arrows). Nevertheless, no skin alterations directly caused by *C. difficile* have ever been reported in patients with CDI. Thus, the effects observed in zebrafish embryos after TcdA administration could be a specific response of the model to the intoxication. 

In recent years, many studies have demonstrated a significant association between low serum HSA levels and CDI development, thus strengthening the hypothesis that hypoalbuminemia predisposes patients to CDI and, in turn, CDI aggravates hypoalbuminemia, generating an auto-sustained vicious cycle [7,8]. Recently, we demonstrated that HSA exerts a protective effect towards CDI, due to HSA capability to bind TcdA and TcdB, thus preventing *C. difficile*-induced host cell damage, both in vitro and in vivo [7,11]. Here, we further investigated HSA ability to neutralize *C. difficile* toxins effect. We observe a significant increase in TcdA+HSA co-treated embryos survival compared to those of animals treated only with TcdA (Figure 8a). Moreover, the results obtained demonstrate that HSA protects zebrafish embryos from both TcdA- or TcdB-dependent extra-intestinal effects, particularly cardiovascular damage and inflammatory response (Figure 8c–e). These results strengthen the idea that HSA may act as a buffer that partially neutralizes the toxins that reach the bloodstream after being produced in the colon. In hypo-albuminemic subjects, in which this buffering activity is impaired, *C. difficile* toxins neutralization in the bloodstream is poorly effective. This, in turn, can contribute to increase clinical severity and poor outcomes associated with CDI.

## 5. Conclusions

We have strengthened the knowledge of the effects of TcdB on the cardiovascular system and we have reported, for the first time, the pathogenic effects of TcdA in the zebrafish embryo model. Moreover, we provided evidences for toxins-dependent effects on the immune system, skin and on the inflammatory response. While further human studies are needed to fully understand the contribution of C. *difficile* toxins to the disease, our data anticipate a significant role for the extra-intestinal effects. Finally, we suggest the potential therapeutic role of HSA to prevent extra-intestinal damage due to *C. difficile* toxins in CDI patients. 

## Supplementary Materials

The following are available online at https://www.mdpi.com/2073-4409/9/12/2575/s1, Figure S1: *C. difficile* toxins affect Caco2 vitality, Figure S2: LPS treatment effects on zebrafish embryos. Table S1: List of primers used in qRT-PCR analysis.

## References

[1] Magill S.S., Edwards J.R., Bamberg W., Beldavs Z.G., Dumyati G., Kainer M.A., Lynfield R., Maloney M., McAllister-Hollod L., Nadle J. (2014). Multistate Point-Prevalence Survey of Health Care—Associated Infections. N. Engl. J. Med..

[2] Leffler D.A., Lamont J.T. (2015). Clostridium difficile Infection. N. Engl. J. Med..

[3] Kuehne S.A., Cartman S.T., Heap J.T., Kelly M.L., Cockayne A., Minton N.P. (2010). The role of toxin A and toxin B in Clostridium difficile infection. Nat. Cell Biol..

[4] Yuan P., Zhang H., Cai C., Zhu S., Zhou Y., Yang X., He R., Li C., Guo S., Li S. (2015). Chondroitin sulfate proteoglycan 4 functions as the cellular receptor for Clostridium difficile toxin B. Cell Res..

[5] Di Bella S., Ascenzi P., Siarakas S., Petrosillo N., Di Masi A. (2016). Clostridium difficile Toxins A and B: Insights into Pathogenic Properties and Extraintestinal Effects. Toxins.

[6] Garey K.W., Sethi S., Yadav Y., Dupont H. (2008). Meta-analysis to assess risk factors for recurrent Clostridium difficile infection. J. Hosp. Infect..

[7] Di Bella S., Di Masi A., Turla S., Ascenzi P., Gouliouris T., Petrosillo N. (2015). The Protective Role of Albumin in Clostridium difficile Infection: A Step Toward Solving the Puzzle. Infect. Control. Hosp. Epidemiol..

[8] Kumarappa V.S., Patel H., Shah A., Baddoura W., Debari V.A. (2014). Temporal changes in serum albumin and total protein in patients with hospital-acquired Clostridium difficile infection. Ann. Clin. Lab. Sci..

[9] Tabak Y.P., Johannes R.S., Sun X., Nunez C.M., McDonald L.C. (2015). Predicting the risk for hospital-onset Clostridium difficile infection (HO-CDI) at the time of inpatient admission: HO-CDI risk score. Infect. Control. Hosp. Epidemiol..

[10] Walker A.S., Eyre D.W., Wyllie D.H., Dingle K.E., Griffiths D., Shine B., Oakley S., O’Connor L., Finney J., Vaughan A. (2013). Relationship between bacterial strain type, host biomarkers and mortality in Clostridium difficile infection. Clin. Infect. Dis..

[11] Di Masi A., Leboffe L., Polticelli F., Tonon F., Zennaro C., Caterino M., Stano P., Fischer S., Hägele M., Müller M. (2018). Human Serum Albumin Is an Essential Component of the Host Defense Mechanism against Clostridium difficile Intoxication. J. Infect. Dis..

[12] Yu H., Chen K., Wu J., Yang Z., Shi L., Barlow L.L., Aronoff D.M., Garey K.W., Savidge T.C., Von Rosenvinge E.C. (2015). Identification of Toxemia in Patients with Clostridium difficile Infection. PLoS ONE.

[13] Jacob S.S., Sebastian J.C., Hiorns D., Jacob S., Mukerjee P.K. (2004). Clostridium difficile and acute respiratory distress syndrome. Heart Lung.

[14] Qualman S.J., Petric M., Karmali M.A., Smith C.R., Hamilton S.R. (1990). Clostridium Difficile Invasion and Toxin Circulation in Fatal Pediatric Pseudomembranous Colitis. Am. J. Clin. Pathol..

[15] Tsourous G.I., Raftopoulos L.G., Kafe E.E., Manoleris E.K., Makaritsis K.P., Pinis S.G. (2007). A case of pseudomembranous colitis presenting with massive ascites. Eur. J. Intern Med..

[16] Mattila P.S., Arkkila P., Tarkka E., Tissari P., Anttila V.-J. (2013). Extraintestinal Clostridium difficile Infections. Clin. Infect. Dis..

[17] Garcáa-Lechuz J., Hernangómez S., Juan R.S., Peláez T., Alcalá L., Bouza E., Lechuz J.M.G. (2001). Extra-intestinal infections caused by Clostridium difficile. Clin. Microbiol. Infect..

[18] Hamm E.E., Voth D.E., Ballard J.D. (2006). Identification of Clostridium difficile toxin B cardiotoxicity using a zebrafish embryo model of intoxication. Proc. Natl. Acad. Sci. USA.

[19] Miller J.D., Neely M.N. (2004). Zebrafish as a model host for streptococcal pathogenesis. Acta Trop..

[20] Westerfield M. (1995). The Zebrafish Book: A Guide for the Laboratory Use of Zebrafish.

[21] Delov V., Muth-Köhne E., Schäfers C., Fenske M. (2014). Transgenic fluorescent zebrafish Tg(fli1:EGFP)y1 for the identification of vasotoxicity within the zFET. Aquat. Toxicol..

[22] Tonon F., Zennaro C., Dapas B., Carraro M., Mariotti M., Grassi M. (2016). Rapid and cost-effective xenograft hepatocellular carcinoma model in Zebrafish for drug testing. Int. J. Pharm..

[23] Zennaro C., Tonon F., Zarattini P., Clai M., Corbelli A., Carraro M., Marchetti M., Ronda L., Paredi G., Rastaldi M.P. (2017). The renal phenotype of allopurinol-treated HPRT-deficient mouse. PLoS ONE.

[24] Novoa B., Bowman T.V., Zon L.I., Figueras A. (2009). LPS response and tolerance in the zebrafish (Danio rerio). Fish Shellfish. Immunol..

[25] Hanke N., Staggs L., Schroder P., Litteral J., Fleig S., Kaufeld J., Pauli C., Haller H., Schiffer M. (2013). “Zebrafishing” for Novel Genes Relevant to the Glomerular Filtration Barrier. BioMed Res. Int..

[26] Zennaro C., Mariotti M., Carraro M., Pasqualetti S., Corbelli A., Armelloni S., Li M., Ikehata M., Clai M., Artero M. (2014). Podocyte developmental defects caused by adriamycin in zebrafish embryos and larvae: A novel model of glomerular damage. PLoS ONE.

[27] Miura G.I., Yelon D. (2011). A guide to analysis of cardiac phenotypes in the zebrafish embryo. Methods Cell Biol..

[28] Sergeeva I.A., Christoffels V.M. (2013). Regulation of expression of atrial and brain natriuretic peptide, biomarkers for heart development and disease. Biochim. Biophys. Acta.

[29] Koenig A.L., Baltrunaite K., Bower N.I., Rossi A., Stainier D.Y., Hogan B.M., Sumanas S. (2016). Vegfa signaling promotes zebrafish intestinal vasculature development through endothelial cell migration from the posterior cardinal vein. Dev. Biol..

[30] Huang J., Kelly C.P., Bakirtzi K., Gálvez J.A.V., Lyras D., Mileto S.J., Larcombe S., Xu H., Yang X., Shields K.S. (2018). Clostridium difficile toxins induce VEGF-A and vascular permeability to promote disease pathogenesis. Nat. Microbiol..

[31] Solomon K. (2013). The host immune response to Clostridium difficile infection. Ther. Adv. Infect. Dis..

[32] Carroll K.C., Bartlett J.G. (2011). Biology of Clostridium difficile: Implications for Epidemiology and Diagnosis. Annu. Rev. Microbiol..

[33] Goonetilleke A., Harris J.B. (2004). Clostridial neurotoxins. J. Neurol. Neurosurg. Psychiatry.

[34] Stevens D.L., Troyer B.E., Merrick D.T., Mitten J.E., Olson R.D. (1988). Lethal effects and cardiovascular effects of purified alpha- and theta-toxins from Clostridium perfringens. J. Infect. Dis..

[35] Naiditch M.J., Bower A.G. (1954). Diphtheria: A study of 1,433 cases observed during a ten-year period at the Los Angeles County Hospital. Am. J. Med..

[36] Trujillo M.H., Castillo A., Espana J., Manzo A., Zerpa R. (1987). Impact of intensive care management on the prognosis of tetanus. Analysis of 641 cases. Chest.

[37] Suffredini D.A., Sampath-Kumar H., Li Y., Ohanjanian L., Remy K.E., Cui X., Eichacker P.Q. (2015). Does Bacillus anthracis Lethal Toxin Directly Depress Myocardial Function? A Review of Clinical Cases and Preclinical Studies. Toxins.

[38] Alhamdi Y., Neill D.R., Abrams S.T., Malak H.A., Yahya R., Barrett-Jolley R., Wang G., Kadioglu A., Toh C.-H. (2015). Circulating Pneumolysin Is a Potent Inducer of Cardiac Injury during Pneumococcal Infection. PLoS Pathog..

[39] Bolz D.D., Li Z., McIndoo E.R., Tweten R.K., Bryant A.E., Stevens D.L. (2015). Cardiac myocyte dysfunction induced by streptolysin O is membrane pore and calcium dependent. Shock.

[40] Musher D.M., Rueda A.M., Kaka A.S., Mapara S.M. (2007). The Association between Pneumococcal Pneumonia and Acute Cardiac Events. Clin. Infect. Dis..

[41] (2018). Buggey J, ElAmm CA: Myocarditis and cardiomyopathy. Curr. Opin. Cardiol..

[42] Van Bruggen N., Thibodeaux H., Palmer J.T., Lee W.P., Fu L., Cairns B., Tumas D., Gerlai R., Williams S.-P., Campagne M.V.L. (1999). VEGF antagonism reduces edema formation and tissue damage after ischemia/reperfusion injury in the mouse brain. J. Clin. Investig..

[43] Fernandez C.E., Bakovic M., Karra R. (2018). Endothelial Contributions to Zebrafish Heart Regeneration. J. Cardiovasc. Dev. Dis..

[44] Harvie E.A., Huttenlocher A. (2015). Neutrophils in host defense: New insights from zebrafish. J. Leukoc. Biol..

[45] Kobayashi Y. (2008). The role of chemokines in neutrophil biology. Front. Biosci..

[46] Yu H., Chen K., Sun Y., Carter M., Garey K.W., Savidge T.C., Devaraj S., Tessier M.E., Von Rosenvinge E.C., Kelly C.P. (2017). Cytokines Are Markers of the Clostridium difficile-Induced Inflammatory Response and Predict Disease Severity. Clin. Vaccine Immunol..

