# Supplementary material for: Extra-Intestinal Effects of C. difficile Toxin A and B: An In Vivo Study Using the Zebrafish Embryo Model
Source: Cells. 2020 Dec 1;9(12):2575. doi: 10.3390/cells9122575 (PMC7760802; doi:10.3390/cells9122575)
Supplement: Supplementary file 1 [file cells-09-02575-s001.zip › Supplementary Figures/Table 1.pdf]

Gene	Primer pair
Nppa	(F) 5’- GAT GTA CAA GCG CAC ACG TT -3’<br>(R) 5’- TCT GAT GCC TCT TCT GTT GC -3’
Nppb	(F) 5’- AAG AGC AGC CCG ATA CTC CTT ACC T -3’<br>(R) 5’- TCC CAA AGA CGA CAT TGA ACC -3’
Vegfa2	(F) 5’- GAT GTG ATT CCC TTC ATG GAT GTG T -3’<br>(R) 5’- GGA TAC TCC TGG ATG ATG TCT ACC A -3’
Flk1	(F) 5’- GAC CAT AAA ACA AGT GAG GCA GAA G -3’<br>(R) 5’- CTC CTG GTT TGA CAG AGC GAT A -3’
Flt1	(F) 5’- AAC TCA CAG ACC AGT GAA CAA GAT C -3’<br>(R) 5’- GCC CTG TAA CGT GTG CAC TAA A -3’
IL1B	(F) 5’- TGG CGA ACG TCA TCC AAG -3’<br>(R) 5’- GGA GCA CTG GGC GAC GCA TA -3’
IL6	(F) 5’- TCA ACT TCT CCA GCG TGA TG -3’<br>(R) 5’- TCT TTC CCT CTT TTC CTC C -3’
CXCL8	(F) 5’- TGT TTT CCT GGC ATT TCT GAC C -3’<br>(R) 5’- TTT ACA GTG TGG GCT TGG AGG G – 3’
Tnfα	(F) 5’- GCT TAT GAG CCA TGC AGT GA -3’<br>(R) 5’- TGC CCA GTC TGT CTC CTT CT -3’
TH	(F) 5’- TTT GAA GAG AAG TGC AGA GGA T -3’<br>(R) 5’- TCA GTA AAT CCT GGG TGA TCC -3’
ACTB	(F) 5’- CAG CAA GCA GGA GTA CGA TGA GT -3’<br>(R) 5’- TTG AAT CTC ATT GCT AGG CCA TT -3’
