# Supplementary figures and images for: Extra-Intestinal Effects of C. difficile Toxin A and B: An In Vivo Study Using the Zebrafish Embryo Model
Source: Cells. 2020 Dec 1;9(12):2575. doi: 10.3390/cells9122575 (PMC7760802; doi:10.3390/cells9122575)

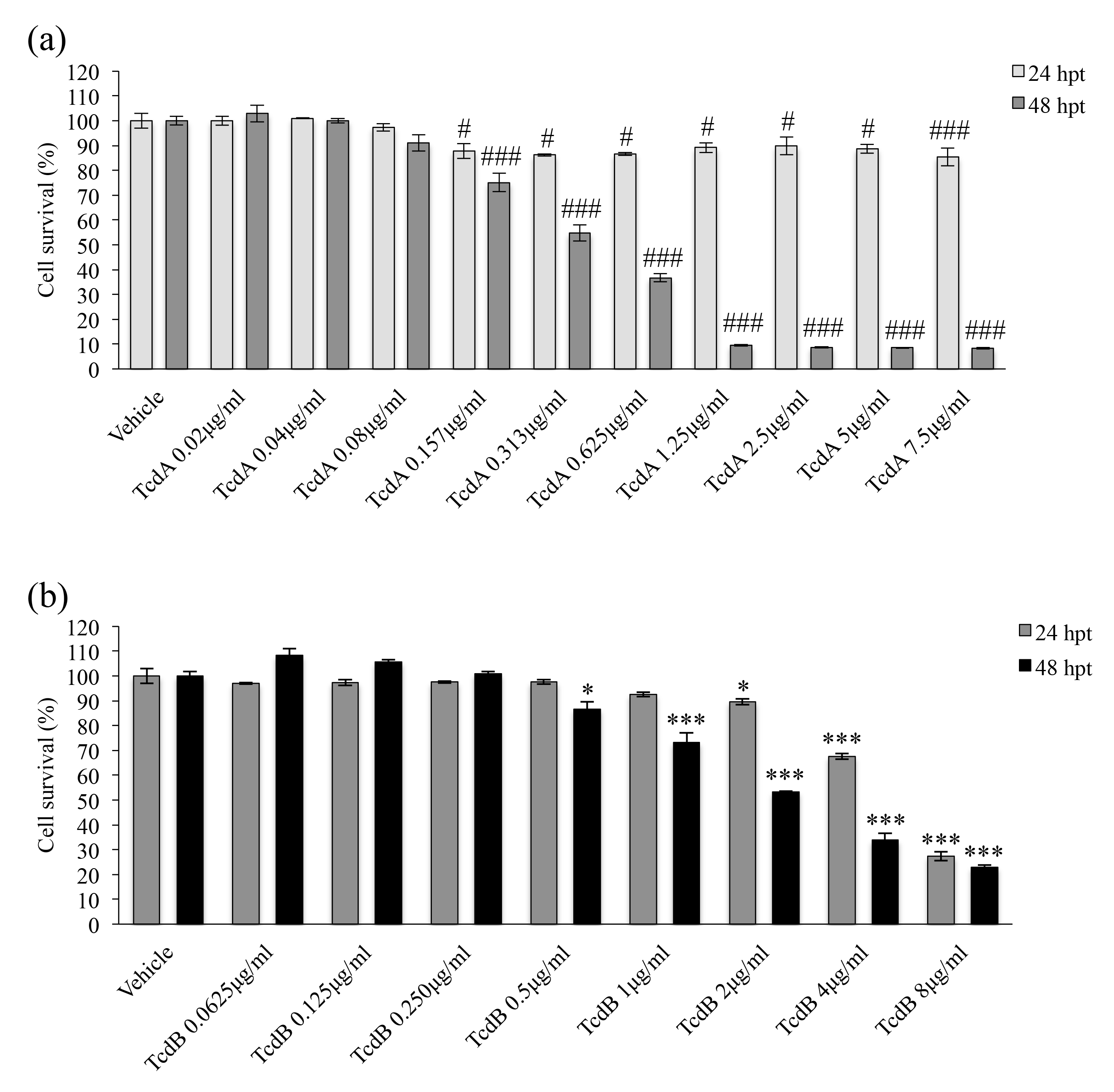

Supplement: Supplementary file 1 [file cells-09-02575-s001.zip › Supplementary Figures/Supplementary Figure 1.tif]

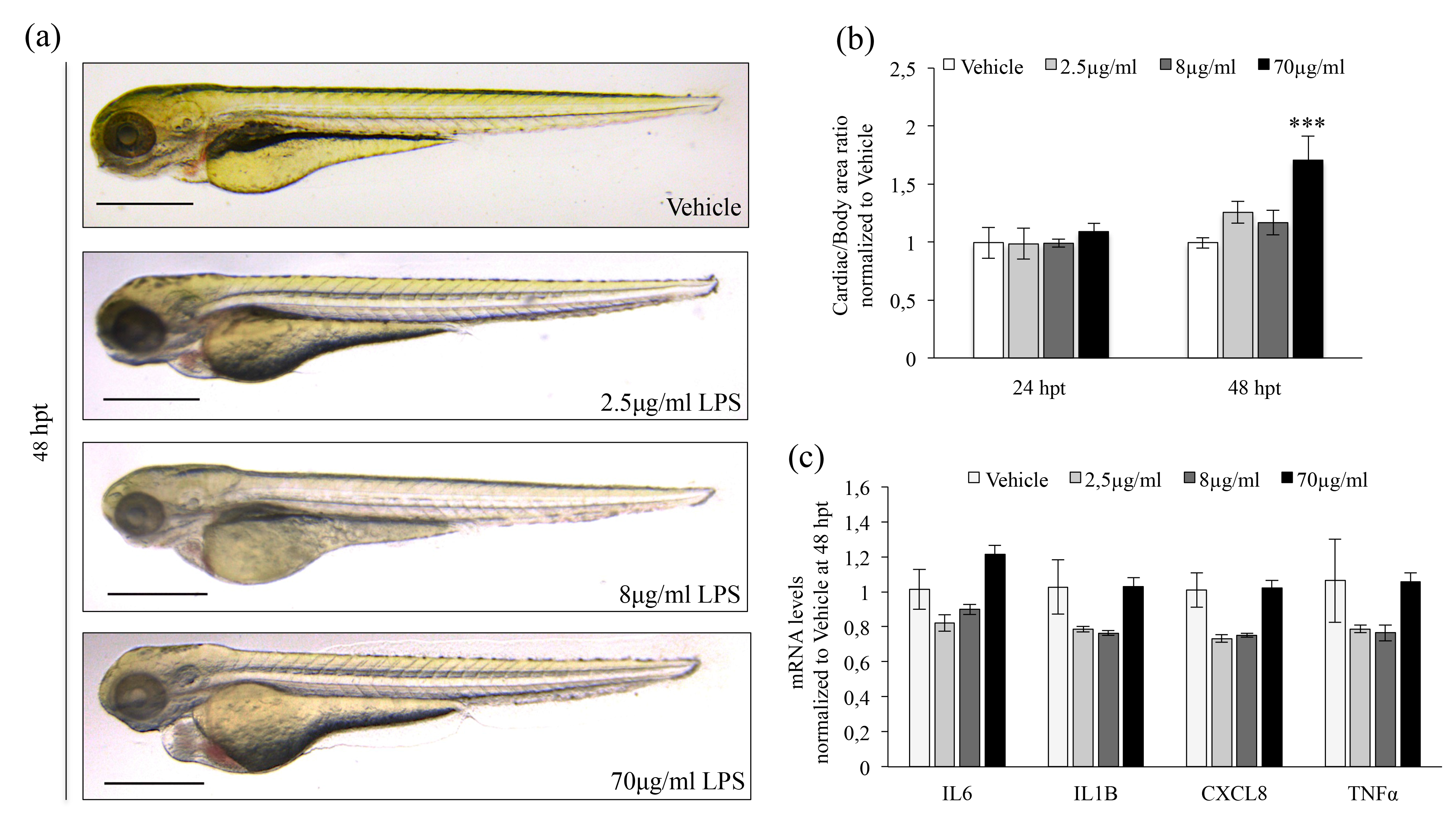

Supplement: Supplementary file 1 [file cells-09-02575-s001.zip › Supplementary Figures/Supplementary Figure 2 26-11-20.tif]
